# Commentary on “Cold scissors ploughing technique versus electrosurgical excision for hysteroscopic adhesiolysis: a multicenter randomized controlled trial”

**DOI:** 10.1097/JS9.0000000000002800

**Published:** 2025-06-25

**Authors:** Jue Zhu, Yichen Chen, Jing Zhang

**Affiliations:** a Department of Gynecology, Women and Children’s Hospital of Ningbo University, Zhejiang, China; b Department of Central Laboratory, Women and Children’s Hospital of Ningbo University, Zhejiang, China

*Dear Editor,*

We read with great interest the recent published by Liu *et al*^[1]^ titled “Cold scissors ploughing technique versus electrosurgical excision for hysteroscopic adhesiolysis: a multicenter randomized controlled trial.” This study, conducted as a multicenter prospective randomized clinical trial, involved the treatment of 217 patients with intrauterine adhesions using either hysteroscopic cold scissors resection or electrosurgical resection. The authors compared changes in AFS scores, menstrual blood loss, recurrence rate, and reproductive outcomes between the cold knife group and the electrosurgical resection group. They found no significant difference in the efficacy of hysteroscopic adhesion release between the two groups, including AFS score changes, recurrence rates, and pregnancy outcomes. This study provides a valuable reference for the selection of surgical instruments related to intrauterine adhesions in clinical practice. However, we believe there are several issues that warrant further clarification and discussion. Our article is compliant with the TITAN Guidelines 2025—governing declaration and use of AI ^[2]^.

First, the inclusion and exclusion criteria set by the authors were not rigorous enough. The inclusion criteria did not specify a requirement for infertility, yet the results indicated a median infertility duration of 2 years among the participants. Previous studies have shown that approximately 43% of patients with intrauterine adhesions experience infertility^[3]^. One possible cause of infertility is the obstruction of the fallopian tubes, uterine cavity, or cervical canal due to adhesions^[3]^. These adhesions may hinder the migration of sperm or the implantation of embryos. It is important to note that not all intrauterine adhesions lead to infertility. In particular, patients with mild intrauterine adhesions have a lower probability of experiencing infertility, and such patients accounted for 17.05% of the study population. If all included patients are to be considered infertile, the authors should clearly specify and exclude common factors related to infertility in the inclusion criteria to accurately assess the pregnancy rate following the treatment of intrauterine adhesions.

Secondly, regarding the estimation of the sample size, the authors based their calculations on the changes in AFS scores between the two groups of patients before and after the operation. However, the final results indicated no significant difference in the changes of AFS scores between the two groups. Therefore, given the current findings, the originally calculated sample size was inadequate for detecting differences between the groups. The premise of sample size calculation relied on the existence of an expected effect, which had not materialized in this case. It may be worthwhile to consider whether other indicators could better reflect the differences between the two surgical methods, allowing for a reevaluation of the sample size.

In addition, regarding the assessment of menstrual blood loss before and after the operation, the authors described that patients evaluated their menstrual blood loss by recording the number of sanitary pads or tampons used, bleeding patterns, and duration of menstruation for one menstrual cycle. They classified the results as increased, no significant change, or decreased. However, there are more accurate methods for assessing menstrual blood loss, such as the Pictorial Blood Loss Assessment Chart (PBAC)^[4]^, which provides a more precise estimation of blood loss rather than simply categorizing it as increased or decreased. Furthermore, the evaluation of blood loss during one menstrual cycle after the operation was conducted under an artificial cycle simulation using estrogen and progesterone. The additional supplementation of estrogen may promote endometrial regeneration, potentially increasing menstrual volume^[5]^. A more accurate assessment should be performed after drug withdrawal. Additionally, the authors did not include measurements of endometrial thickness, which can simultaneously reflect menstrual volume and therapeutic effect, thereby reducing the reliability of the study.

Finally, in the context of intrauterine adhesion surgery, the primary challenge lies with patients who have severe intrauterine adhesions. The cure rate after surgery for mild to moderate intrauterine adhesions was reported to be 86.96%^[6]^, while for severe adhesions, the recurrence rate may reach 62.5%^[7]^. However, only 18 patients (8.29%) with severe adhesions were included in this study. Due to this limited sample size, the authors were unable to conduct further stratified analyses. As a result, the differences and relative effectiveness of the two surgical methods for severe intrauterine adhesions could not be fully assessed, thereby limiting the conclusions drawn from the study.

In conclusion, we acknowledge the clinical significance of the research and analysis conducted by the authors. This study provides a useful basis for clinicians in choosing between cold knife resection and electroresection for the treatment of intrauterine adhesions. However, we suggest that the authors further improve the research design and refine the methodology in future studies to enhance the reliability and clinical applicability of their findings.

## Data Availability

Not applicable.
